# Allergy to polyethylene glycol and polysorbates in a patient cohort: Diagnostic work‐up and decision points for vaccination during the COVID‐19 pandemic

**DOI:** 10.1002/clt2.12111

**Published:** 2022-01-08

**Authors:** Charlotte G. Mortz, Henrik F. Kjaer, Trine H. Rasmussen, Helene M. Rasmussen, Lene Heise Garvey, Carsten Bindslev‐Jensen

**Affiliations:** ^1^ Department of Dermatology and Allergy Centre, Odense Research Centre for Anaphylaxis (ORCA), Odense University Hospital University of Southern Denmark Odense C Denmark; ^2^ Allergy Clinic Copenhagen University Hospital at Gentofte Copenhagen Denmark

**Keywords:** Anaphylaxis, COVID‐19 vaccines, excipients, polyethylene glycols, polysorbate

## Abstract

**Background:**

During the COVID‐19 pandemic focus has been on polyethylene glycol (PEG) and polysorbate as these excipients are constituents in the first vaccines and possible elicitors of allergic reactions to the vaccines. We aimed to evaluate the possibility of vaccinating patients with PEG and/or polysorbate allergy against COVID‐19.

**Methods:**

Twenty‐five patients with a history of an allergic reaction to drugs, vaccines and mouth hygiene products containing PEG or polysorbate and sensitization (skin test or in vitro test) or a positive challenge were included. We re‐evaluated 19 of 21 patients diagnosed before 2021 and four new patients by skin prick tests (SPT) and Basophil Histamine Release (BaHR) for PEGs, polysorbates and approved COVID‐19 vaccines as well as measurement of specific IgE (PEG 2000, 10,000). Patients were offered vaccination based on decision points from the primary diagnosis and re‐evaluation.

**Results:**

Most common primary elicitors were depot‐steroids and laxatives. Most patients had experienced more than one reaction. SPT was superior to BaHR test although many SPTs became negative over time. After careful re‐evaluation three patients were successfully vaccinated with the Pfizer/BioNTech vaccine. Three were vaccinated before referral. Eleven were offered the Johnson‐Johnson vaccine; four were vaccinated successfully, seven abstained. Six patients could not be vaccinated with PEG or polysorbate containing vaccines.

**Conclusion:**

Hypersensitivity to excipients in COVID‐19 vaccines constitutes a risk to patients with allergy to PEG or polysorbates. After diagnostic evaluation, a safe COVID‐19 vaccine could be offered to most patients, the remainders will await new vaccines containing different excipients.

## 1 INTRODUCTION

In March 2020 the World Health Organization (WHO) declared a pandemic for severe acute respiratory syndrome coronavirus type 2 (SARS‐CoV‐2)—also called COVID‐19.^1^ Vaccines were developed with unprecedented speed as a major global preventive measure against COVID‐19, and since December 2020 many million doses of various vaccines have been administered globally. In general, severe allergic reactions to vaccines are rare and caused by either the vaccine itself or by excipients contained in the vaccine.^2^, ^3^, ^4^

Excipients are necessary to support and optimize the properties of the active ingredients in vaccines, drugs, and other products. Immediate‐type hypersensitivity has been described to several excipients with most reports on polyethylene glycols (PEGs) also called macrogols (E1521)^5^ and the structurally related polysorbates.^6^ During the COVID‐19 epidemic focus has been on these two excipients as they are excipients in the first vaccines on the market in Europe and possible culprits for allergic reactions to the COVID‐19 vaccines.^7^, ^8^, ^9^, ^10^

Polyethylene glycols are used in pharmaceutical and medical products including vaccines, as well as in cosmetics and industrial and food products.^5^ PEGs are polymers of ethylene oxide with molecular weights (MW) ranging from 200 to 35,000 g/mol. In drugs and other pharmaceutical products, the PEG numbers are described by the average MW, but in cosmetics PEGs are described by the average number of ethylene oxide units.^5^ Absorption occur through the gastro‐intestinal mucosa^11^, ^12^ and intact skin^5^ depending on MW.

Polyethylene glycol allergic patients may show cross‐reactions to PEGylated drugs and structurally similar polymers such as polysorbates and poloxamers. However, the evidence for this still is sparse with only a few cases described.^6^, ^13^, ^14^

Polysorbate 80 (E433), also called tween 80, is used in drug formulations including vaccines, as well as in food and cosmetics as a solubilizer, stabilizer or emulsifier.^15^ It is a fatty acid ester of polyoxyethylene‐sorbitan. Other polysorbates include polysorbate 20 and 60.

PEG 2000 is used in the mRNA vaccines from Pfizer/BioNTech (Comirnaty^®^) and Moderna (Spikevax^®^) and polysorbate 80 in viral vector vaccines from several other companies including AstraZeneca (Vaxzevria^®^) and Johnson‐Johnson (Janssen vaccine^®^). In Denmark, only the COVID‐19 vaccines from Pfizer/BioNTech (PB) and Moderna (M) are recommended by the Danish Medicines Agency. The AstraZeneca (AZ) vaccine was withdrawn from the market in March 2021 due to safety concerns and the Johnson‐Johnson (JJ) vaccine was never approved. However, the highly specialized allergy centers in Denmark have gained permission from the Danish Health Authority to offer the JJ vaccine to patients with PEG allergy based on a medical assessment and risk evaluation including the risk of severe COVID‐19 disease, the risk of severe side effects including vaccine‐induced immune thrombotic thrombocytopenia (VITT) and the risk of an allergic reaction to the vaccine.

We have recently published results on anaphylactic reactions to COVID‐19 vaccines in the normal population and found the incidence to be similar to other virus‐based vaccines.^10^ Of 55 patients with reactions to the first dose 52 patients were re‐vaccinated without adverse reactions. Three patients were diagnosed with allergy to drug excipients.^10^

EAACI has recently published a statement on the diagnosis, management and prevention of severe allergic reactions to COVID‐19 vaccines^16^ stating that unless the patient has a history of an allergic reaction to any of the vaccine components, there is no contraindication to administer the currently approved COVID‐19 vaccines to allergic patients. However, knowledge and guidance on how to safely evaluate and vaccinate patients allergic to COVID‐19 vaccine excipients is still missing.

We here describe the allergological work‐up program leading to a decision for safe COVID‐19 vaccination in patients with a history of an allergic reaction to drugs, vaccines and mouth hygiene products containing PEG or polysorbate and sensitization or positive challenge to one or more of these excipients.

## 2 METHODS

### 2.1 Population (*n* = 25)

From August 01, 2015 to December 31, 2020 a total of 21 adults were diagnosed with PEG and/or polysorbate allergy at the Allergy Center, Odense University Hospital. The diagnosis was based on a positive case history to a culprit drug/agent containing PEG or polysorbate combined with a positive skin prick test (SPT), BaHR (Histamine Release) test and/or a positive challenge to one or more PEGs or polysorbates. From January 01, 2021 to May 01, 2021 further four patients were identified. All 25 patients consented to registration in the Allergy Center Database, Odense University Hospital, Denmark. Two patients were lost to follow‐up for the re‐evaluation in 2021.

### 2.2 Primary evaluation at diagnosis 2015–2020 (*n* = 21) and in 2021 (*n* = 4)

A detailed case history was obtained including comorbidity, suspected culprit drugs/agents and WAO grading of anaphylaxis severity^17^ for the first/most severe reaction.

At the primary evaluation SPT to a panel of excipients was performed. SPT was performed with a 1 mm ALK Lancet at the volar surface of the forearm. Histamine solution (10 mg/ml) and saline were used as positive and negative control respectively.^18^, ^19^ The size of the resulting wheals was recorded after 15 and 30 min and wheal size was measured on the longest and shortest perpendicular axis, numbers were added and divided by two to obtain the mean wheal diameter. Wheals ≥3 mm larger than the negative control were considered positive. The SPT panel included PEG 300 (100%), 400 (50%), 3000 (50%), 3350 (100%), 6000 (50%), PEG 20,000 (0.01%, 0.1%, 1%, 10%, 20%, stepwise with 30 min interval until a positive response), polysorbate 20 (100%), polysorbate 80 (100%) and poloxamer 407 (10%). Excipients were prepared in sterile water and received from the laboratory of Medical Allergology, Gentofte Hospital, Hellerup, Denmark (PEG 300, 3000, 6000, 20,000, Poloxamer 407)^20^ and from the laboratory at the Allergy Center, Odense University Hospital [PEG 400, 3000, 6000 (Merck), PEG 3350 (Movicol junior Neutral^®^, Norgine B.V.), polysorbate 80 and 20 (Merck)].

A Histamine Release (BaHR) test (www.Reflab.dk) was performed using the same allergens as in SPT.

All challenges were performed in a titrated protocol as part of standard operating procedures at the Allergy Center. Movicol^®^ (PEG 3350) was used as a marker for PEG.

### 2.3 Re‐evaluation in relation to COVID‐19 vaccination (2021) (*n* = 23)

From January 2021 the patients were offered further testing including SPT and BaHR test with the COVID‐19 vaccines (and their purified excipients) on the Danish market. Repeated SPT with PEGs, polysorbates and poloxamer 407 was performed to re‐assess sensitization. Skin testing was performed in duplicate with the available vaccines and their excipients prepared at the laboratory at the Allergy Center, Odense University Hospital; Pfizer/BioNTech (PB) vaccine, Moderna (M) vaccine, AstraZeneca (AZ) vaccine (using residual remnants of the original vials, obtained daily from our in house vaccination center for hospital staff), PEG 2000 (Thermo Fisher, concentration 50%), DMG‐PEG 2000 (Merck, concentration 20%), ALC‐0159 PEG 2000 (Sinopeg, China, concentration 20%), and Polysorbate 80 (Merck, concentration 100%). Furthermore, re‐test with PEG 6000, 3000 (Merck, concentration 50%), PEG 3350 (Movicol junior Neutral^®^, Norgine B.V., concentration 100%), and PEG 300, Polysorbate 20 (Merck, concentration 100%). PEG 20,000 (concentration 0.01%, 0.1%, 1%, 10%, 20%, titrated, not in duplicate) and Poloxamer 407 (10%) was obtained from the laboratory of Medical Allergology, Gentofte Hospital, Hellerup, Denmark.^20^

Furthermore, specific IgE to PEG 2000 and PEG 10,000 was measured in 2021 (Thermo Fisher Scientific) using ImmunoCAP (https://dfu.phadia.com) with the modification of replacing the standard wash buffer with alternative detergent (*n*‐octyl‐beta‐d‐glucopyranoside solution) to exclude tween (polysorbate 80) from the standard wash buffer.

### 2.4 Criteria for selection of a possible COVID‐19 vaccine (*n* = 20)

In 2021 the excipient allergy status for each patient was re‐classified as certain, possible or unlikely based on primary history, primary testing, the course and re‐evaluation and based on this, criteria were made for selection of a safe COVID‐19 vaccine whenever possible.

All COVID‐19 vaccinations were performed by giving the full dose in anaphylaxis surveillance with IV access and observation for at least 2 h. For patients receiving the Johnson‐Johnson (JJ) vaccine a SPT in duplicate with JJ vaccine was performed 30 min before the vaccination.

### 2.5 Ethics

All included patients were registered in the Allergy Center Database, and oral and written informed consent was obtained from all patients. The study was approved by the Danish Data Protection Agency (Journal nr.: 20/63311) and the Ethics Committee (Report nr.: Covid ‐ 21/209, nr. 50).

## 3 RESULTS

### 3.1 Primary evaluation at diagnosis 2015–2020 (*n* = 21) and in 2021 (*n* = 4)

In total, 25 patients with a diagnosis of PEG allergy and/or polysorbate allergy were included (Table S1), five males and 20 females, mean age at diagnosis 42.4 years (range 19–84 years).

The most common eliciting drugs were depot steroids followed by laxatives (Table S1 and Table 1). More than half of the patients had experienced more than one reaction to drugs, vaccines or mouth hygiene products (14/25). Five patients reacted to new tablets even after the diagnosis had been established (three analgesics, two antidepressants). Seven of the 25 reported local reactions to topical products containing the excipients, all were females (Table S1).

**TABLE 1:** Culprit drugs, vaccines and mouth hygiene products at first and subsequent reactions among 25 patients with sensitization or positive challenge to polyethylene glycol and/or polysorbate

	Elicitor at first reaction	Total numbers of reactions among the 25 patients
Depot‐steroid injections	10	15
Laxatives (PEG)	5	7
Vaccines	2	3
Analgesic tablets	1	6
Antibiotic tablets	1	1
Antacids	1	1
PPI	1	1
Antihistamine tablets	1	1
Antidepressant tablets	0	2
Estradiol vagitory	0	1
TNF alfa infusion	0	2
Nose spray	1	3
Mouth hygiene products (toothpaste, mouthwash)	2	5
Total	25	46

Twenty patients had reactions to drugs, vaccines and consumer products containing PEG, but no history of reactions to polysorbate, while four patients described reactions to both PEG and polysorbate containing products (ID12, ID14, ID16, ID24). The excipient in a depot steroid was unknown in one patient (ID5).

Twelve patients fulfilled the WAO criteria for anaphylaxis for at least one reaction with WAO score 5 in five patients, WAO score 4 in two and WAO score 3 in five. Five patients had a WAO score 2, and eight patients a WAO score 1, of these three experienced delayed reactions with generalized urticaria after 12–24 h.

The diagnostic delay was up to 8 years; however, in 17/25 patients the diagnosis was established within 1 year. Co‐morbidity is shown in Table S1.

At the primary evaluation (*n* = 25) allergy was concluded based on a certain case history and positive SPT in 17 patients, in 6 by a certain or possible case history and positive BaHR (negative SPT in 5/6, no SPT in 1/6) and two with a certain case history were negative in both SPT and BaHR test but were positive on oral challenge with PEG 3350. A total of six challenges with PEG 3350 were performed between 2015 and 2020 (Table S1). Furthermore, ID16 diagnosed in 2021 with polysorbate allergy, had a PEG 3350 challenge due to a positive SPT to PEGs without a history of reaction to PEG containing products and PEG challenge was positive.

### 3.2 Re‐evaluation of patients diagnosed 2015–2020 (*n* = 21; two lost to follow up) in relation to COVID‐19 vaccination (2021)

Reproducibility of SPT and BaHR to PEGs and polysorbates over time was assessed in 19 patients diagnosed 2015–2020 and re‐tested in 2021.

Twelve patients had a positive SPT to PEGs at the diagnosis. Table 2 shows the reproducibility of SPT over time. At the re‐examination, only six (orange in Table 2) of the 12 patients originally testing positive on SPT were positive in 2021. Of the six patients testing negative on SPT two developed urticaria after the SPT (yellow in Table 2), one was BaHR positive (gray in Table 2), while three were both SPT and BaHR negative (white in Table 2). The time between the first and second testing was 1–3 years in those still positive in SPT and 2–6 years in the other groups.

**TABLE 2:** Re‐evaluation of 12 patients with a previous positive skin prick test to polyethylene glycol and polysorbate in 2015–2020 (orange still SPT positive, yellow negative second SPT but urticaria after second SPT, gray negative second SPT but BaHR positive, white no reactions)

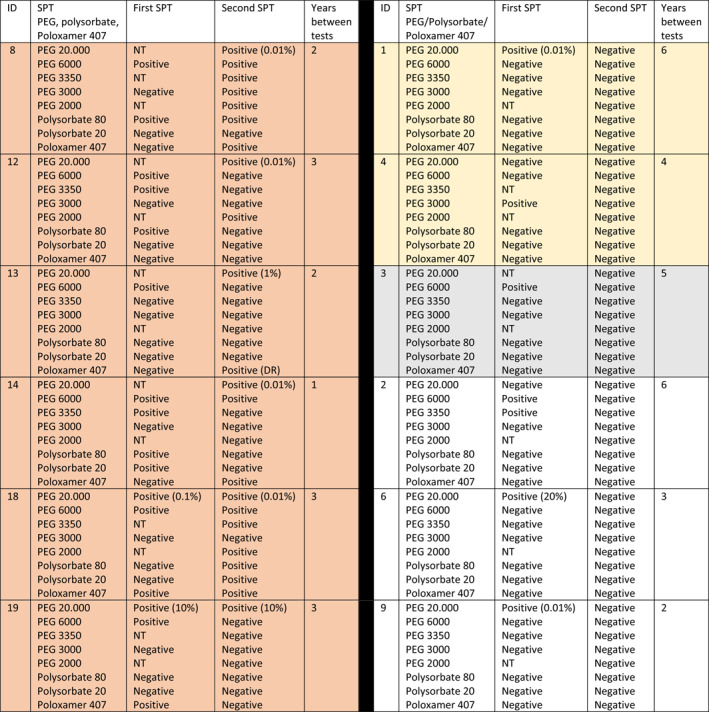

*Note*: Specific IgE > 0.10 in 2021: ID8, 12, 18.

Abbreviations: DR, delayed reaction; NT, not tested.

Of the two (ID7, ID11) with negative SPT and BaHR, but a positive oral challenge at the primary evaluation, one had become BaHR positive to polysorbate 80 of unknown relevance, the other reacted with urticaria 1 h after SPT.

All five patients, only positive in BaHR at the primary evaluation, were negative in both SPT and BaHR.

### 3.3 SPT results for COVID‐19 vaccines and their excipients in 2021 for 19 patients diagnosed 2015–2020 and four patients diagnosed 2021 (*n* = 23); see Table 3

SPT to the Moderma (M) vaccine was positive in two patients, one of whom also had a positive SPT to the AstraZeneca (AZ) vaccine; both also had a positive SPT to PEG 2000, DMG‐PEG 2000 and polysorbate 80 as well as other PEGs and polysorbate 20. One of these patients (ID16) had a primary reaction to the AZ vaccine, the other (ID18) had a previous reaction to several drugs containing PEG (Table S1). No reactions to the Pfizer/BioNTech (PB) vaccine were seen in SPT although it was the only vaccine tested in all 23 patients. Among the 23 patient assessed, in total seven had a positive SPT reaction to one or more of the PEG 2000 variants and 5 to polysorbate 80.

### 3.4 IgE results in 2021 (*n* = 23)

Specific IgE (sIgE) to PEG 2000 and 10,000 were measured and were higher than 0.10 kIU/L in four patients for PEG 2000 and in five for PEG 10,000 (Table 3). All four with elevated values for PEG 2000 had positive SPT to PEGs, however, the clinical history varied from mild reactions (ID8, ID10) to moderate (ID18) and severe anaphylaxis (ID12).

**TABLE 3:** Results of COVID‐19 related testing for vaccines, polyethylene glycols and polysorbates at re‐evaluation in 2021 and vaccine offered based on risk evaluation

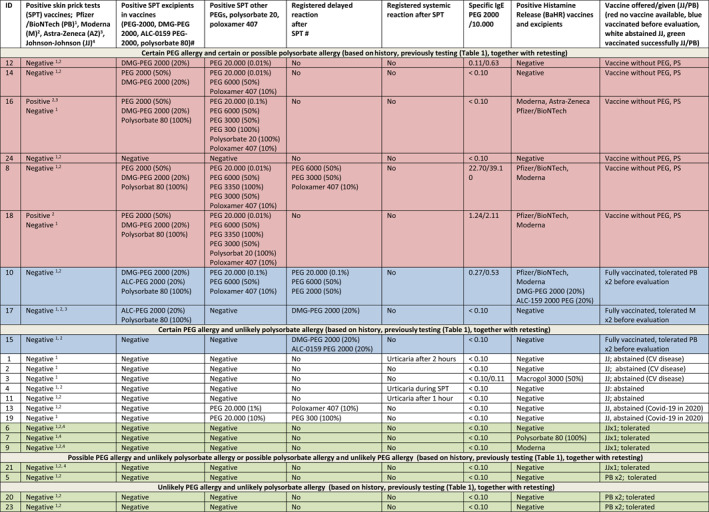

*Note*: From Table S1 ID 22, 25 were lost to follow up and are therefore not included. # ALC‐0159 PEG 2000 only tested in ID 10, 15, 16, 17.

Abbreviations: CV, cardiovascular; PS, polysorbate.

### 3.5 Re‐evaluation of diagnosis in 2021 and selection of patients for COVID‐19 vaccine (*n* = 23, three already fully vaccinated); see Table 4 and Figure 1

**FIGURE 1:**
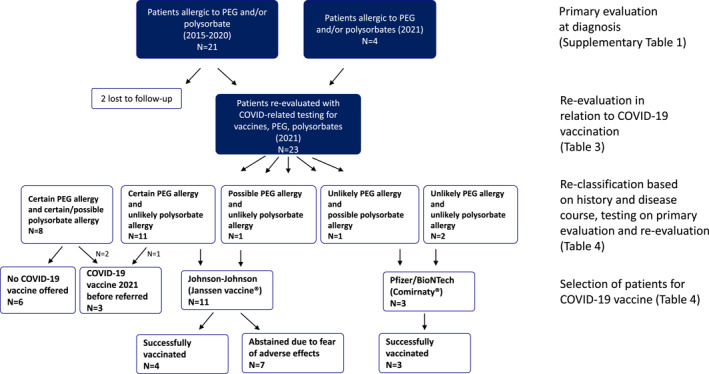
Flow charge of the evaluation of the included patients from primary evaluation to re‐evaluation and re‐classification in 2021 and selection of COVID‐19 vaccine

The allergy status of the patients was re‐classified in 2021 as certain, possible and unlikely based on history and disease course, testing on primary evaluation (Table S1) and re‐evaluation (Tables 2 and 3). The decision to offer COVID‐19 vaccination was made after a risk evaluation based on this information (Table 4, Figure 1).

**TABLE 4:** Criteria for risk evaluation related to COVID‐19 vaccine (*n* = 23, two lost to follow up)

Group	History and testing	Covid‐19 vaccine offered	Number of patients
Certain PEG allergy and certain polysorbate allergy	Certain history of both PEG and polysorbate allergy	No PEG or polysorbate vaccine	4
	+ sensitization to PEG or polysorbate or both		
Certain PEG allergy and possible polysorbate allergy	Certain history of PEG allergy and PEG sensitization + no history of polysorbate allergy but present SPT sensitization to polysorbate + elevated sIgE PEG	No PEG or polysorbate vaccine	2
Certain PEG allergy and unlikely polysorbate allergy	Certain history of allergy to PEG and not polysorbate	Polysorbate vaccine (JJ)	10 offered; 3 accepted
	+ sensitized/challenge positive to PEG not polysorbate		
Possible PEG allergy and unlikely polysorbate allergy	Possible history of allergy to PEG and not polysorbate	Polysorbate vaccine (JJ)	1 offered; 1 accepted
	+ sensitized/challenge positive to PEG not polysorbate		
Possible polysorbate allergy and unlikely PEG allergy	Possible history of allergy to polysorbate and not PEG	PEG vaccine (PB)	1 offered; 1 accepted
	+ sensitized/challenge positive to polysorbate not PEG		
Unlikely PEG allergy and unlikely polysorbate allergy	Uncertain history of PEG allergy, no history of polysorbate allergy + negative SPT and sIgE, and transient positive BaHR to a single PEG ± polysorbate	PEG vaccine (PB)	2 offered; 2 accepted
Fully vaccinated, reaction after second dose			3

Abbreviations: JJ, Johnson‐Johnson; PB, Pfizer/BioNTech; PEG, polyethylene glycol.

The four patients with a certain history of both PEG and polysorbate allergy and sensitization to PEG or polysorbate or both were not offered a present COVID‐19 vaccination in Denmark [ID12, ID14, ID16 and ID24 (red in Table 3)].

Two patients (ID8, ID18) had a certain history of allergy to PEG and PEG sensitization as well as present sensitization to polysorbate (possible allergy) and elevated specific IgE to PEG 2000 (red in Table 3) and these were not offered vaccination either.

Ten patients had a certain PEG allergy but unlikely polysorbate allergy, and were offered a JJ vaccine. Three accepted (ID6, ID7, ID9, green in Table 3) and were successfully vaccinated while seven abstained due to fear of adverse effects (ID1‐4, ID11, ID13, ID19, white in Table 3).

After re‐evaluation ID21 was classified as possible PEG allergy unlikely polysorbate allergy and was successfully JJ vaccinated (green in Table 3). ID5 had a possible polysorbate allergy and unlikely PEG allergy (challenge negative to PEG 3350 in 2021) and ID20 and ID23 had unlikely PEG and polysorbate allergy. All three were were offered PB vaccine and vaccinated successfully twice at the Allergy Center (green in Table 3).

Three patients were fully vaccinated before they were referred in 2021 and the decision for re‐vaccination in these patients will await the vaccine status at the time of the re‐vaccination (blue in Table 3).

## 4 DISCUSSION

There is an urgent need for an allergological work‐up program in patients with allergy to PEG and/or polysorbates to make decisions for safe COVID‐19 vaccinations. Presently, PEG‐2000 is used in the mRNA vaccines from PB and M and polysorbate 80 in viral vector vaccines from several other companies including from AZ and JJ.

We here present a cohort of 25 patients with a history of allergic reactions to drugs, vaccines and consumer products containing PEG or polysorbate and sensitization or positive challenge to the excipient in the period 2015–2021. After thorough allergological work‐up including SPT and BaHR with PEGs, polysorbates and vaccines supplemented with challenges when needed, the allergy status was classified in 2021 as certain, possible or unlikely based on the primary evaluation (Table S1), disease course from diagnosis until re‐testing and re‐testing in 2021 (Table 3). Using risk evaluation criteria based on the gathered information (Table 4) it was possible to offer COVID 19 vaccination to 14 patients out of 20 evaluated (Figure 1). An additional three patients were fully vaccinated (2 PB, 1 M) prior to evaluation.

Only six patients could not be offered vaccines containing PEG or polysorbate due to certain clinical reactions to both excipients. Ten patients with certain PEG allergy and one with possible PEG allergy, all 11 with unlikely polysorbate allergy, were offered a JJ vaccine; four accepted and were vaccinated without reaction. One patient with unlikely PEG allergy and possible polysorbate allergy and two with unlikely PEG and polysorbate allergy were offered the PB vaccine and all three received both doses uneventfully. This illustrates, that patients with reactions and sensitizations to PEGs but no clinically relevant reactions or sensitizations to polysorbate 80 can be safely vaccinated with a polysorbate containing vaccine. The role of SPT sensitization to polysorbates in PEG allergic patients has to be evaluated further. For safety reasons we did not offer a polysorbate vaccine in those with present PEG allergy including elevated specific IgE and polysorbate sensitization. Isolated polysorbate allergy is rare, but if sensitization to PEGs could be excluded, a PEG containing vaccine could be considered under anaphylaxis surveillance. An additional challenge in Denmark is, that the viral vector vaccine from JJ and AZ are not recommended by the Danish health authorities due to the risk of thrombotic side effects. The population are therefore not so likely to accept an offer of these vaccines and seven out of 11 patients offered the JJ vaccine abstained; three due to cardio‐vascular disease and four decided to wait for new vaccines (two had already had COVID‐19 infection).

In our cohort 12 (48%) fulfilled the WAO criteria for anaphylaxis (grade 3–5) at diagnosis; six were treated with adrenalin. Thirteen patients had WAO scores 1–2, most of those had reactions with generalized acute urticaria/angioedema. Most primary reactions were elicited by depot‐steroid injections followed by laxatives (Table 1) as also reported in other studies.^5^, ^6^, ^14^, ^21^ More than half (14/25) of the patients have had more than one reaction. Most (11/25) had all the reactions prior to the excipient allergy diagnosis being established supporting the previously described delay in diagnosis in this patient group, who are also at risk of being misdiagnosed as idiopathic anaphylaxis, urticaria or allergy to the active ingredient in the drug.^14^, ^21^, ^22^ Despite careful information five also had reactions after the diagnosis was established highlighting that the allergy is very challenging for the patients. Most health care workers and pharmacists are not aware of this allergy and special expertise and comprehensive follow‐up by an allergist with special knowledge on excipient allergy is needed.^14^ As the content of PEGs and polysorbates may vary in different formulations of the same drug each individual drug needs to be checked for excipients. Furthermore, labeling of excipients in drugs and consumer products varies and different names are used. After the diagnosis had been established, seven women reported mild skin reactions such as localized erythema or urticaria to topical products containing PEG or polysorbates. This has also recently been shown in another Danish study where re‐exposure reactions mainly were elicited by everyday cosmetic products used on the skin and with mild reactions as also found in our patient group.^14^ Is it unknown why more women than males (20/25) were identified in our cohort. However, one possible explanation could be that the primary sensitization occur through the skin and women are in generally more exposed to topical products than males. However, it is unknown if the primary sensitization is through the gastro‐intestinal tract or the skin. A mean age of 42 years could indicate that a longer exposure time is needed before sensitization.

In our cohort, when comparing SPT at diagnosis with SPT at re‐evaluation in 2021, half of the patients had lost their skin test reactivity (Table 2), however some reacted with systemic urticaria after the SPT. One patient who had become negative in SPT was included in a previous publication from our group,^22^ then the patient was tested three times over 1 year and after 1 year the SPT turned negative despite a positive challenge to PEG 3350 and a clear cut history and primary evaluation. A recent Danish paper examining skin test reactivity to PEGs over time also showed that patients with longer interval since diagnosis tested negative to lower MW PEGs and positive mainly to higher concentrations of PEG 20,000.^20^ This means that the timing of allergy testing is important to get reliable results as also seen for other drug allergens.^23^, ^24^

The sensitivity, specificity and predictive values of SPT and BaHR test in relation to excipient allergy have not been established. In our cohort BaHR seems to be a poor marker for PEG and polysorbate allergy. We used the published concentrations in SPT for PEGs, polysorbates and poloxamer except for polysorbate 80, where we used 100% instead of 20%.^20^, ^25^ This may result in false positive, irritative reactions or in worst case anaphylaxis. However, we only have few positive SPT reactions to polysorbate 80 in 100% (9/25) in our cohort and none of these patients had severe reactions to skin testing. Testing in another cohort without history of excipient allergy in our department in 2021 (*n* > 500) no positive reactions to polysorbate 80 in 100% were seen (unpublished data).

Only two patients tested positive to the COVID 19 vaccines; both also tested positive to the vaccine excipient. More tested positive to the vaccine excipients, but negative to the vaccines themselves. The concentrations of excipients in the vaccines are unknown, and testing with the vaccines seems not to give additional information in our cohort.

The immunological mechanisms in PEG anaphylaxis are not clear but an IgE mediated mechanism has been suggested.^26^, ^27^, ^28^ Specific IgEs to PEG 2000 were higher than 0.10 in four patients (Table 3). All had a positive SPT to at least one of the three different PEG 2000 tested. The IgE positive patients did not all have a severe history to PEG, two of four had anaphylaxis but two others had generalized urticaria. The value of this specific IgE to PEG 2000 should be further studied.

As PEG may elicitate anaphylaxis even by SPT, oral challenge is only indicated if the allergy is not verified by a very suggestive history and skin test sensitization. The only products on the Danish market declaring the concentration of PEG 3350 is a laxative, Movicol^®^. Although not knowing the fraction absorbed from the GI tract, we performed titrated oral challenges to verify or rule out allergy in those with possible allergy. We had no severe reactions by challenge.

In summary, evaluation for excipient allergy is challenging; the reproducibility of SPT test is time dependent and reactivity will decline over time, while BaHR seems to be a poor marker. In selected cases a challenge can be performed if case history and sensitization pattern is not clear. Hypersensitivity to excipients in COVID‐19 vaccines constitute a risk to patients with allergy to PEG or polysorbates. To our knowledge there is no publications on COVID‐19 vaccination in these patients. After diagnostic evaluation, we could offer a safe COVID‐19 vaccine to most patients, and all who accepted were vaccinated without any allergic symptoms. The remaining patients will have to await new vaccines containing different excipients or for some patients fractionated vaccination could be considered in anaphylaxis surveillance at an Allergy Center as is currently initiated in our center.

## CONFLICT OF INTEREST

The authors report no potential conflict of interest.

## Supporting information

Table S1
