# Supplementary material for: Allergy to polyethylene glycol and polysorbates in a patient cohort: Diagnostic work‐up and decision points for vaccination during the COVID‐19 pandemic
Source: Clin Transl Allergy. 2022 Jan 8;12(1):e12111. doi: 10.1002/clt2.12111 (PMC8742452; doi:10.1002/clt2.12111)
Supplement: Supplementary file 1 — Table S1 [file CLT2-12-e12111-s001.docx]

Supplementary Table 1:

Culprit(s), clinical manifestations, co-morbidity and test results at primary evaluation of the 25 patients

| **ID** | **Age** | **Sex** | **Culprit drug/agents**  **(bold after diagnosis)** | **Time from reaction to diagnosis** | **WAO grade first/ most severe reaction and symptoms**  **+ Adrenaline** | **Local**  **reaction to**  **topical**  **products** | **Positive SPT excipients at diagnosis** | **Positive BaHR excipients at diagnosis** | **Challenge**  **Movicol (PEG 3350)** | **Other test results** | **Co-morbidity** |
| --- | --- | --- | --- | --- | --- | --- | --- | --- | --- | --- | --- |
| 1 | 57 | M | Diprospan steroid injection (PEG 3350)  Depo-Medrol steroid injection (PEG 3350)  Moviprep laxative (PEG 3350)  **Escitalopram (PEG 400)** | 8 y | WAO 5, **+ Adrenaline**  Generalized urticaria and angioedema  Dyspnea  Hypotension | None | PEG 20.000 (0.01%)  Generalized urticaria a few hours after SPT | Negative | Positive IR | Negative SPT steroids, negative challenge Solu-Medrol  SPT, IgE chlorhexidine and latex negative | Depression  Back, knee and shoulder problems  CV disease  No atopic disease |
| 2 | 45 | F | Depo-Medrol steroid injection (PEG 3350)  **Panodil Zapp (PEG 6000)** | < 1 y | WAO 4, **+ Adrenaline**  Generalized urticaria  Throat tightness, dyspnea, wheezing | None | PEG 6000 (50%)  PEG 3350 (100%) | Negative | Positive IR | Negative SPT steroids, negative challenge Solu-Medrol, lidocaine. SPT, IgE chlorhexidine and latex negative | CV disease  Achilles tendinitis  No atopic disease |
| 3 | 64 | F | Depo-Medrol steroid injection (PEG 3350) | <1 y | WAO 5  Flushing, pruritus soles  Dyspnea  Hypotension | None | PEG 6000 (50%)  PEG 20.000 NT | Negative | Negative  – low dose | Negative SPT steroids, negative challenge Solu-Medrol, lidocaine  SPT, IgE chlorhexidine and latex negative | Rheumatoid arthritis  CV disease  No atopic disease |
| 4 | 47 | F | Diprospan steroid injection (PEG 3350)  **Panodil Zapp (PEG 6000)** | 3 y | WAO 1  Delayed reaction with face erythema and generalized urticaria after 24 hours | None | PEG 3000 (50%)  Polysorbate 80 (100%)  PEG 3350 NT | Negative | Positive DR with urticaria  In 2021# | Negative SPT steroids, Diprospan challenge with delayed reaction  SPT, IgE chlorhexidin and latex negative | Dermatitis  Hip problems  No atopic disease |
| 5 | 57 | F | Unidentified depot steroid injection | >3 y | WAO 3  Flushing  Dyspnea | None | Negative  PEG 3350 NT | Polysorbate 20 (100%) | Negative  In 2021 # | Negative SPT steroids, negative challenge Solu-Medrol, lidocaine  SPT, IgE chlorhexidine and latex negative | Rheumatoid arthritis  Kidney cancer antea  No atopic disease |
| 6 | 46 | F | Depo-Medrol steroid injection (PEG 3350) | < 1 y | WAO 2  Angioedema  Rhinitis  Conjunctivitis | None | PEG 20.000 (20%) | PEG 6000 (100%)  PEG 3000 (50,100%) | ND | Negative SPT steroids, negative challenge Solu-Medrol, lidocaine  SPT, IgE chlorhexidine and latex negative | Arthritis  No atopic disease |
| 7 | 25 | M | Depo-Medrol steroid injection (PEG 3350) x 2 | 3 y | WAO 1  Generalized urticaria | None | None | Negative | Positive IR | Negative SPT steroids, negative challenge Solu-Medrol  IgE chlorhexidine and latex negative | Allergic rhinitis  Schizophrenia |
| 8 | 50 | F | Diprospan steroid injection (PEG 3350)  Movicol laxative (PEG 3350)  Vagifem (PEG 6000) | < 1 y | WAO 1  Generalized urticaria | Wash powder (PEG?) | PEG 6000 (50%)  Polysorbat 80 (100%)  PEG 3350, 20.000 NT | PEG 20.000 (10%) | ND | IgE Chlorhexidine and latex negative | Asthma  Allergic rhinitis |
| 9 | 34 | F | Depo-Medrol steroid injection (PEG 3350) x3 | < 1 y | WAO 2  Rhinitis with sneezing  Nausea | Cream  (PEG 100) | PEG 20.000 (0.1%) | PEG 6000 (50%)  PEG 3000 (50%)  PEG 20.000(10%) | ND | Negative SPT steroids, negative challenge Solu-Medrol, lidocain  IgE Chlorhexidine and latex negative | Reactive arthititis  No atopic disease |
| 10 | 47 | F | Depo-Medrol steroid injection (PEG 3350) | <1 y | WAO 1  Generalized urticaria | None | PEG 20.000 (0.1%)  PEG 6000 (50%)  DMG-PEG 2000 (20%)  ALC-PEG 2000 (20%)  PEG 2000 (after 6 hours)  Poloxamer 407 (10%)  Polysorbate 80 (100%) | Negative | ND | Negative SPT steroids, chlorhexidine and latex.  IgE Chlorhexidine and latex negative | Diabetes (insulin pump)  Frozen shoulder  No atopic disease |
| 11 | 21 | F | Movicol laxative (PEG 3350) | < 1 y | WAO 2  Generalized urticaria  Nasal congestion, sneezing, throat tightness | Creme  (PEG 40) | Negative | Negative | Positive IR | SPT, IgE Klorhexidin and latex negative | No atopic disease |
| 12 | 38 | M | Movicol laxative (PEG 3350)  Infliximab (polysorbate 80) x2 | <1 y | WAO 5  Pruritus palms and soles  Hypotension  Loss of consciousness | None | PEG 6000 (50%)  PEG 3350 (100%)  Polysorbate 80 (100%)  PEG 20.000 NT | Negative | ND | IgE Chlorhexidine and latex negative | Asthma  Colitis ulcerosa |
| 13 | 34 | F | Movicol laxative (PEG 3350) | <1 y | WAO 5  Generalized urticaria  Hypotension | None | PEG 6000 (50%)  PEG 20.000 NT | PEG 6000 (50%) | ND | IgE Chlorhexidine and latex negative | No atopic disease |
| 14 | 31 | F | Moxaxole laxative (PEG 3350)  **Brintellix (PEG 400)** | <1 y | WAO 2  Flushing, itching in the palms  Generalized angioedema  Rhinitis (sneezing, secretion) | Self tan (PEG)  Make-up (poly-sorbate) | PEG 6000 (50%)  PEG 3350 (100%)  Polysorbate 80 (100%)  Polysorbate 20 (100%)  PEG 20.000 NT | Negative | ND | IgE Chlorhexidine and latex negative | Migraine  No atopic disease |
| 15 | 59 | F | Moviprep laxative (PEG 3350)  Vaccine Pfizer/BioNTech (PEG 2000) – 2. dose | 5 y | WAO 2  Generalized urticaria  Nausea | None | DMG-PEG 2000 (20%)  ALC-0159 PEG 2000(20%)  Both after 2 h | Negative | ND | IgE Chlorhexidin and latex negative | CV disease  Cervical hematoma  Bursitis  No atopic disease |
| 16 | 19 | F | Astra-Zeneca vaccine (Polysorbate 80) – 1. dose | <1 y | WAO 1  Delayed reaction with generalized urticaria after 12 h | None | PEG 20.000 (0.1%)  PEG 6000 (50%)  PEG 3000 (50%)  PEG 2000 (50%)  DMG-PEG 2000 (20%)  PEG 300 (100%)  Poloxamer 407 (10%)  Polysorbate 80 (100%)  Polysorbate 20 (100%) | Negative | Positive IR in 2021# | IgE Chlorhexidin and latex negative | CSU (mild, no daily treatment)  Allergic contact dermatitis eyedrops  No atopic disease |
| 17 | 88 | F | Moderna vaccine (PEG 2000)- first dose | <1 y | WAO 1  Delayed reaction with generalized urticaria after 12 h | None | ALC-PEG 2000 (20%)  Polysorbate 80 (100%)  DMG-PEG 2000 (20%); after 2 hours | Negative | ND | sIgE Chlorhexidin and latex negative  Vaccination with second dose Moderna without reaction, positive SPT thereafter | Aortastenosis  Hypertension arterialis  Hypercholesteromi  No atopic disease |
| 18 | 22 | F | Primcillin (PEG 6000)  Depo-Medrol steroid injection (PEG 3350) | 3 y | WAO 3, **+ Adrenaline**  Generalized urticaria and angioedema  Nausea  Throat tightness  Dyspnea, cough | Face cream (PEG)  Steroid-cream (PEG) | PEG 20.000 (0.1%)  PEG 6000 (50%)  Poloxamer 407 (10%)  PEG 3350 NT | Poloxamer 407 (10%) | ND | Negative SPT steroids  IgE Chlorhexidin and latex negative  Lidocaine challenge negative.  Positive challenge Primcillin with PEG | Allergic rhinitis  ASA/NSAID intolerance  Ankle problems |
| 19 | 30 | F | Lanzoprazol (PEG 6000)  **Panodil Zapp (PEG 6000)** | <1 y | WAO 5, **+ Adrenaline**  Flushing, pruritus palms and soles Generalized urticaria and angioedema  Hypotension  Loss of consciousness | Cream (PEG 100)  Washing powder (PEG?) | PEG 20.000 (10%)  PEG 6000 (50%)  Poloxamer 407 (10%)  PEG 3350 NT | Negative | ND | Negative SPT, HR lanzoprazol  SPT, IgE Chlorhexidine and latex negative | No atopic disease |
| 20 | 24 | F | Telfast (PEG 400) | 7 y | WAO 3, **+ Adrenaline**  Generalized urticaria and angioedema  Dyspnea, throat tightness | None | Negative  PEG 3350 NT | PEG 300 (100%)  Polysorbate 80 (100%) | ND | Negative SPT and challenge Telfast  IgE latex and chlorhexidine negative | CSU (mild, no daily treatment)  No atopic disease |
| 21 | 60 | F | Paracetamol x3 (PEG) | < 1y | WAO 1  Severe pruritus | None | Negative  PEG 3350 NT | PEG 6000(50%)  PEG 3000(50%)  Poloxamer 407 (10%) | ND | SPT and challenge with culprit paracetamol negative  SPT, IgE latex and chlorhexidine negative | ASA/NSAID intolerance  Penicillin allergy  Dyspepsia  No atopic disease |
| 22 | 31 | M | Balancid (PEG 6000) | < 1y | WAO 4, **+ Adrenaline**  Generalized urticaria, angioedema  Dyspnea, wheezing | None | Negative  PEG 3350 NT | PEG 3000 (50%) | Negative  – low dose | SPT and HR positive Gaviscon | CV disease  Gaviscon allergy  No atopic disease |
| 23 | 40 | M | Colgate toothpaste x2 (PEG) | < 1y | WAO 3  Angioedema  Dyspnea | None | Negative  PEG 3350 NT | PEG 6000 (50%) | ND | SPT, HR negative culprit  SPT, IgE latex and chlorhexidine negative. Use Colgate in 2021. | Neopren allergy  Cerebral aneurysm  No atopic disease |
| 24 | 28 | F | Oral B tandpasta (PEG)  Aquafresh tandpasta (PEG) Colgate mouth-wash (polysorbate 20) | <1 y | WAO 3  Generalized urticaria  Dyspnea | Cream (poly-sorbate 60) | NT | Polysorbate 80 (100%) | ND | IgE chlorhexidine and latex negative  Food allergy evaluation negative | CSU  Hypothyroidism  NSAID intolerance  No atopic disease |
| 25 | 63 | F | Zymelin noose spray menthol x3 (PEG) | < 1y | WAO 1  Generalized urticaria, angioedema | None | PEG 3000 (50%)  Polysorbate 20 (100%) | Negative | ND | SPT, HR and challenge negative Zymelin noose spray | Allergic rhinitis |

Notes: ID 10, 15, 16, 17 evaluated for the first time in 2021, ID 22 and 25 lost to follow up. # Challenge in relation to re-evaluation in 2021. Abbreviations: PEG: Polyethylene glycol. F: Female, M: Male. Y: Year, NT: Not tested. ND: Not done. SPT: Skin prick test. HR: Histamine release. IgE: Immunoglobuline E. CV: Cardiovascular, CSU: Chronic spontaneous urticaria.
